# Positive Expiratory Pressure Improves Oxygenation in Healthy Subjects Exposed to Hypoxia

**DOI:** 10.1371/journal.pone.0085219

**Published:** 2013-12-23

**Authors:** Hugo Nespoulet, Thomas Rupp, Damien Bachasson, Renaud Tamisier, Bernard Wuyam, Patrick Lévy, Samuel Verges

**Affiliations:** 1 INSERM U1042, Grenoble, France; 2 University Grenoble Alpes, Hypoxia-Physiopathology Laboratory (HP2), Grenoble, France; Vanderbilt University Medical Center, United States of America

## Abstract

**Introduction:**

Positive end-expiratory pressure (PEEP) is commonly used in critical care medicine to improve gas exchange. Altitude sickness is associated with exaggerated reduction in arterial oxygenation. We assessed the effect of PEEP and pursed lips breathing (PLB) on arterial and tissue oxygenation under normobaric and hypobaric hypoxic conditions.

**Methods:**

Sixteen healthy volunteers were exposed to acute normobaric hypoxia (Laboratory study, FiO_2_=0.12). The protocol consisted in 3-min phases with PEEPs of 0, 5 or 10 cmH_2_O, PLB or similar ventilation than with PEEP-10, interspaced with 3-min phases of free breathing. Arterial (pulse oximetry) and quadriceps (near-infrared spectroscopy) oxygenation, ventilation, cardiac function, esophageal and gastric pressures and subjects’ subjective perceptions were recorded continuously. In addition, the effect of PEEP on arterial oxygenation was tested at 4,350 m of altitude in 9 volunteers breathing for 20 min with PEEP-10 (Field study).

**Results:**

During the laboratory study, PEEP-10 increased arterial and quadriceps oxygenation (arterial oxygen saturation +5.6±5.0% and quadriceps oxyhemoglobin +58±73 µmol.cm compared to free breathing; p<0.05). Conversely, PLB did not increase oxygenation. Oxygenation improvement with PEEP-10 was accompanied by an increase in expiratory esophageal and gastric pressures (esophageal pressure swing +5.4±3.2 cmH_2_O, p<0.05) but no change in minute ventilation, breathing pattern, end-tidal CO_2_ or cardiac function (all p>0.05) compared to PEEP-0. During the field study, PEEP-10 increased arterial oxygen saturation by +6.7±6.0% after the 3^rd^ minute with PEEP-10 without further significant increase until the 20^th^ minute with PEEP-10. Subjects did not report any significant discomfort with PEEP.

**Conclusions:**

These data indicate that 10-cmH_2_O PEEP significantly improves arterial and muscle oxygenation under both normobaric and hypobaric hypoxic conditions in healthy subjects. PEEP-10 could be an attractive non-pharmacological tool to limit blood oxygen desaturation and possibly symptoms at altitude.

## Introduction

Pursed-lips breathing (PLB) is commonly used in order to reduce dyspnea and to improve gas exchange in patients with chronic obstructive pulmonary disease [1,2]. Positive end-expiratory pressure (PEEP) is also used in critical care medicine to improve pulmonary gas exchange and compliance [3,4]. At least part of the effect of PEEP and PLB on gas exchange is thought to result from an increase in alveolar pressure, leading to improved lung diffusion capacity and blood oxygenation [5,6]. At high-altitude or in long-time flight, the decrease in barometric pressure induces a significant hypoxemia in mountaineers and travelers. As a consequence of hypoxemia, acute mountain sickness (AMS) and pulmonary or cerebral edema may occur and put one’s life at risk [7]. It is well known that the severity of these pathologies is closely correlated with the degree of arterial oxygen desaturation (S_p_O_2_). Burtscher et al. [8] showed that, for a given altitude (>2,500 m) or equivalent normobaric hypoxic level, a difference of about 4.9% of S_p_O_2_ is a key factor that distinguishes people who develop symptoms of altitude intolerance and those remaining clinically healthy. Peripheral O_2_ chemosensitivity appears to be a critical factor involved in this S_p_O_2_ difference [9,10]. On field, acetazolamide and theophylline are commonly used in order to prevent or treat AMS, because of their ability to increase hypoxic ventilatory response and improve S_p_O_2_. In addition to increased ventilation, improved pulmonary diffusion is another mechanism associated with altitude acclimatization and enhanced blood oxygenation [11]. Therefore, any intervention that might improve pulmonary diffusion could potentially increase blood oxygenation and altitude tolerance.

Using positive expiratory pressure as a method to improve arterial oxygenation at altitude has been suggested by some authors [11–15], but its efficiency and the potential mechanisms involved remain to be clarified. In particular, no study has been able to verify whether the improvement in oxygen saturation while increasing expiratory pressure is associated with changes in minute ventilation or breathing pattern [16]. Increased tidal volume [14] and minute ventilation (V_E_) [15] as previously reported with PEEP may induce *per se* some improvement in blood oxygenation [15]. Various levels of PEEP have been used (5 cmH_2_O [13,14], 7 cmH_2_O [11], 10 cmH_2_O [15]) but no comparison has been done between different PEEP levels to determine the optimal pressure. The individual tolerance to the device also remains to be investigated. In addition, PEEP is known to significantly modify cardiac function [17] but heart rate (HR) only has been measured during PEEP in hypoxia and inconsistent changes have been reported [11,13,15]. Hence, it remains to assess whether PEEP during hypoxic exposure induces significant changes in left ventricular stroke volume (SV) and cardiac output (Qc).

PLB, which does not require any equipment, can also increase tidal volume, minute ventilation and oxygen saturation in healthy subjects or in patients [2,18,19]. A recent field case study assessed the effect of PLB in a subject presenting severe AMS at 4,330 m [20]. A rapid and critical increase in S_p_O_2_ has been observed but no other parameter was measured. Hence, whether PLB may lead to similar effects than PEEP on blood oxygenation during hypoxic exposure remains to be investigated. 

To clarify the effects of PEEP and PLB in healthy subjects during hypoxic exposure, we tested the following hypotheses: i) PEEP improves S_p_O_2_ and muscle oxygenation in hypoxia irrespective of any change in ventilation; ii) PEEP level (0 *versus* 5 *versus* 10 cmH_2_O) has a significant impact on oxygenation improvement; iii) PLB is as efficient as PEEP in improving oxygenation under hypoxic conditions; iv) PEEP improves S_p_O_2_ under both normobaric and hypobaric hypoxic conditions. In addition, we aimed to evaluate the effect of PEEP and PLB on pleural pressure, cardiac function and subjects’ subjective perception.

## Materials and Methods

### Subjects

Sixteen healthy male volunteers (age: 38.1 ± 11.5 yrs, BMI: 22.1 ± 1.8 Kg·m^-2^) participated in the laboratory study and 9 additional healthy male volunteers (age: 31.2 ± 11.9 yrs, BMI: 23.4 ± 2.0 Kg·m^-2^) participated in the field study. All participants gave their informed written consent, and the study was approved by the local ethics committee (CPP Sud-Est V, Grenoble). All subjects were natives of low altitude and none stayed at altitude above 3,000 m during the two months before the study. All were free of cardiovascular, pulmonary or neurological diseases. They all had normal lung function (forced vital capacity: 110 ± 11% predicted; forced expiratory volume in 1 s: 109 ± 12% predicted) and normal lung diffusion capacity (alveolar capillary membrane conductance: 97 ± 16% predicted; pulmonary capillary blood volvolume: 83 ± 15% predicted; alveolar volvolume: 109 ± 24% predicted) [21]. The laboratory study was conducted in the Grenoble University Hospital (210 m of altitude; barometric pressure, Pb = 740 mmHg) while the field study was conducted at the Observatoire Vallot near the Mont-Blanc, France (4,350 m; Pb = 448 mmHg). 

### Experimental protocol

During the laboratory study, subjects were seated comfortably in a semi-supine position and were connected with a mouthpiece and a three-way valve to an ergospirometric device measuring gas exchange and breathing pattern (SensorMedics, Yorba-Linda, Ca, USA). The inspiratory side of the valve was connected to an Altitrainer® (SMTEC, Nyon, Switzerland) delivering a gas mixture with an inspiratory oxygen fraction (F_i_O_2_) of 0.12 (normobaric hypoxia, equivalent to 4,300 m of altitude; inspiratory oxygen pressure, PiO_2_ = 83 mmHg). The expiratory side of the valve was adapted to fit with a mechanical resistance (Ambu PEEP®, 0-10 cmH_2_O, Ballerup, Danmark). S_p_O_2_ was continuously measured at the finger with a Nonin 4100 oxymeter (Nonin Medical, Plymouth, MN). After 20 min of quiet breathing in hypoxic conditions in order to stabilize arterial desaturation, subjects had to breathe for successive phases of 3 min with specific conditions of ventilation, 3 min of free breathing (FB, no PEEP device) separating each condition. Five conditions were tested: a PEEP of 0 cmH_2_O (PEEP-0), 5 cmH_2_O (PEEP-5), 10 cmH_2_O (PEEP-10), breathing at the ventilatory level spontaneously reached with PEEP-10 (the target ventilation was continuously coached by the experimenter to the subject) but with no PEEP device (T10) and PLB. The PEEP-0 condition was used as a sham condition since subjects were blinded for the PEEP level. For the PLB condition, subjects were trained to perform this technique before the experimental session to avoid any learning effect during the protocol. They were asked to breathe using a short inhalation followed by a long exhalation against a resistance induced by pursed lips that would induce no major breathing discomfort. The image of “breathing through a straw” was used. Within 30 min, all five conditions (PEEP-0, PEEP-5, PEEP-10, T10 and PLB) were performed in a random order (except that T10 was always performed after PEEP-10). This 30-min sequence was repeated twice, the whole experimental session lasting for 80 min. Ventilation, esophageal and gastric pressures, cardiac function, quadriceps oxygenation and subjects’ subjective perceptions were continuously measured during the laboratory study (see below).

 The field study was performed following the laboratory study to assess the effect of PEEP during hypobaric hypoxic exposure (after 2.9 ± 1.5 days at 4,350 m following helicopter ascent, i.e. after the initial 2 days of acclimatization; PiO_2_ = 84 mmHg) on SpO_2_. Subjects were seated comfortably and breathed with a mouthpiece through a three-way valve that was connected on the expiratory side to the same device as during the laboratory study inducing a PEEP of 10 cmH_2_O. After 3 minutes of FB, subjects breathed for 20 min with PEEP-10 while S_p_O_2_ was continuously measured at the finger (Nonin Medical).

### Measurements

#### Esophageal and gastric pressures

In order to estimate thoracic (pleural) and abdominal pressures and the work of breathing, esophageal (P_es_) and gastric (P_ga_) pressures were measured by conventional balloon catheters (Milic-Emili et al. 1964), connected to pressure transducers (model DP45-30, Validyne, Northridge, CA). The pressure analog signal was digitized (MacLab, ADInstruments, Castle Hill, Australia) and recorded on a computer (Chart Software version 5.0, ADInstruments).

#### Cardiac function

SV, HR and Qc were measured using a non-invasive impedance cardiography device (Physioflow®, Manatec Biomedical, Paris, France). Physioflow is an impedance technique based on the principle that variations in the impedance to a high-frequency (75 kHz) low-magnitude (1.8 mA) alternating current across the thorax during cardiac ejection result in a waveform from which SV can be calculated. Initially, SV index is calculated at rest by evaluating 24 consecutive heart beats (autocalibration procedure), using measurements of the largest impedance difference during systole, as well as the largest rate of variation of the impedance signal (contractility index), the thoracic fluid inversion time, HR, and pulse pressure (i.e. the difference between systolic and diastolic arterial pressure) [22]. Cardiac output is then calculated by multiplying the SV index with body surface area and HR [22].

After cleaning the skin, two pairs of electrodes (FS50, Skintact, Innsbruck, Austria) were positioned at the left base of the neck and along the xiphoid for transmitting and receiving electrical currents. Two electrodes were also placed on the chest (V1/V6 position) to obtain the ECG signal. The autocalibration procedure was started after a period of at least 5 min, in which patients were immobile. SV, HR and Qc values were stored beat-to-beat. 

#### Pulmonary function tests

Lung function and lung diffusion capacity for carbon monoxide and nitric oxide (T_L_CO/NO) measurements were performed in a BodyBox 5500 (Medi-Soft, Dinant, Belgium). For T_L_CO and T_L_NO, procedures and normal values were those reported by Aguilaniu et al. [21], from which transfer coefficient, membrane conductance, capillary volume and alveolar volume were derived.

#### Skeletal muscle oxygenation

Muscle oxygenation was measured continuously by near-infrared spectroscopy (NIRS) [23] with a four-wavelength (775, 810, 850, 905 nm) high temporal resolution NIRS device (NIRO-300, Hamamatsu Photonics, Hamamatsu City, Japan). NIRS probes were attached to the skin on the lower third of the belly of the right *vastus lateralis* (range of 15–20 cm above the proximal border of the patella) and in parallel with the long axis of the muscle. The distance between the transmitting and receiving optodes was fixed at 4 cm by a probe holder secured to the skin using double-sided tape and covered with a black sweatband maintained with an elastic muff net to shield the optodes from ambient light.

Data were collected with a sampling frequency of 2 Hz. For each phase, relative concentration changes (∆μmol·cm) of oxy-(∆[O_2_Hb]), deoxy-(∆[HHb]) and total (∆[THb] = [O_2_Hb] + [HHb]) hemoglobin were measured from the previous FB condition. We used a multidistance spatially resolved tissue oximeter (NIRO-300) that is able to quantify tissue oxy-hemoglobin saturation directly as a tissue oxygenation index (TOI) reflecting the dynamic balance between O_2_ supply and O_2_ consumption in the investigated muscle volume [23].

#### Subject’s perception

Subjects indicated their subjective perception of well-being and breathing effort on two visual analog scales (VAS) of 100-mm long. At the beginning and at the end, there were labeled with ‘perfect well-being’ on the left end and ‘unbearable condition’ on the right end or ‘no breathing effort’ on the left end and ‘extreme breathing effort’ on the right end. Subjects were carefully instructed and asked to score their subjective perception of well-being from ‘as before starting the study’ (i.e. ‘perfect well-being’) to ‘it cannot be even worse” (i.e. ‘unbearable condition’) and ‘how difficult is it to breathe from ‘no difficulty’ (i.e. ‘no breathing discomfort’) to ‘extremely difficult’ (i.e. ‘extreme breathing discomfort’). The subject’s marks on the 100-mm VAS were converted in scores between 0 and 10. Both VAS were filled during the last 30 s of each condition during the whole experiment. 

### Data analysis

For the laboratory study, all measurements represent the mean value of the last 30 s of each condition. Because no significant difference was observed for any breathing condition between the first and the second 30-min period, data presented for each breathing condition (PEEP-0, PEEP-5, PEEP-10, T10 and PLB) are the averaged value from the two repetitions of each 3-min phases. Because no significant difference was observed between all FB conditions, FB values are the average of all FB conditions. The comparison of parameters between the different breathing conditions (FB, PEEP-0, PEEP-5, PEEP-10, T10 and PLB) was achieved using one-way analysis of variance (ANOVA) with repeated measurements. When significant main effects were found, Fischer’s p-tests were used for post hoc analysis.

For the field study, changes in SpO_2_ over time (averaged for 3 min of FB and for each minute with PEEP-10) were assessed with a one-way analysis of variance (ANOVA) with repeated measurements and Fischer’s p-tests for post hoc analysis. 

All statistical calculations were performed on standard statistics software (Statview 5.0, SAS Institute, Cary, North Carolina). Significance was set at p < 0.05. All descriptive statistics presented as mean values ± SD.

## Results

### Laboratory study

#### Changes in *S*_p_O_2_

**Figure 1:**
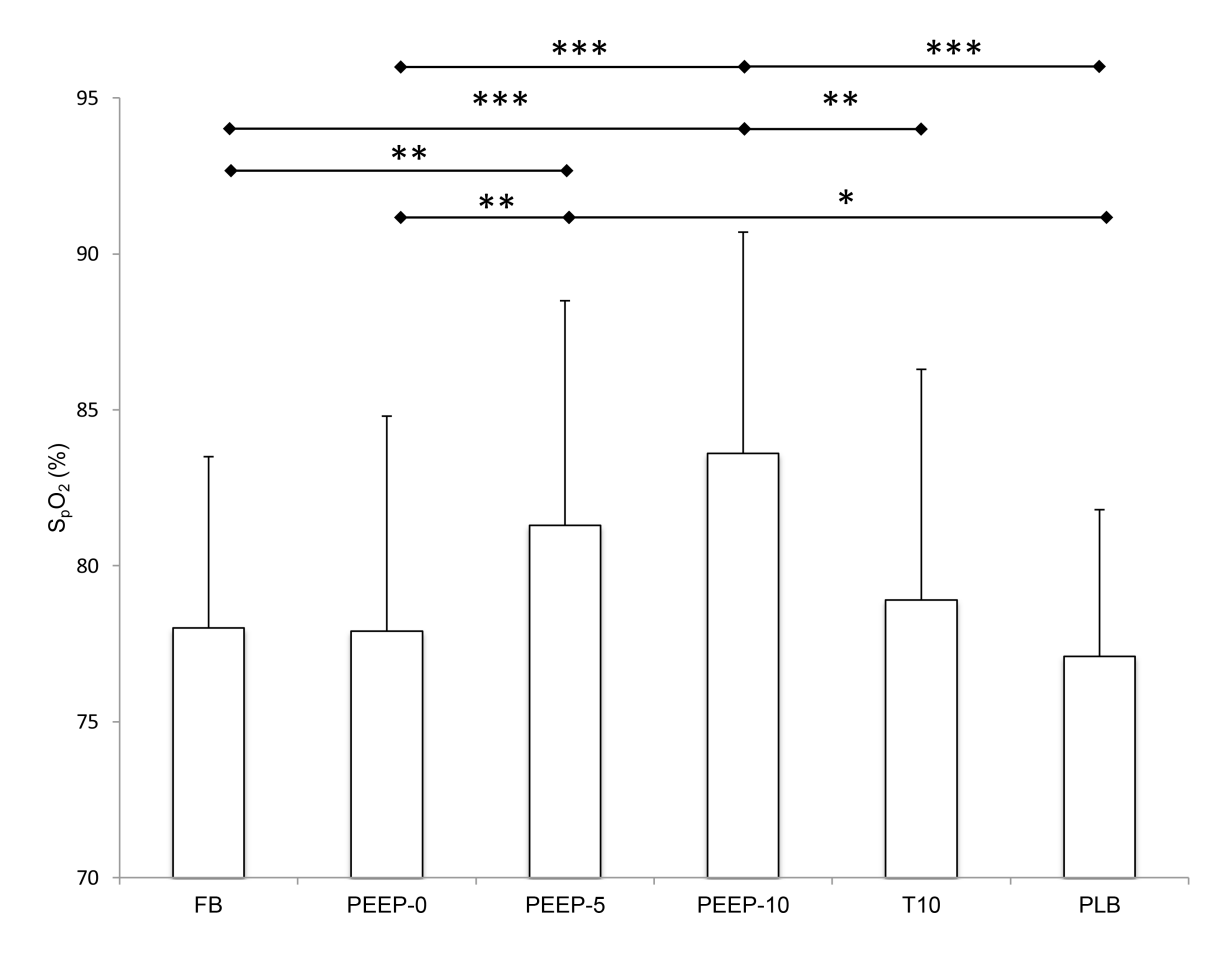
Arterial blood oxygen saturation (S_p_O_2_) in all experimental conditions. FB, free breathing with no PEEP device; PEEP-0/5/10, Positive end expiratory pressure at 0, 5 and 10 cmH_2_O; T10, same ventilatory level as measured in PEEP-10 condition but without PEEP device; PLB, pursed lips breathing. * significant difference between two conditions (* p < 0.05, **, p < 0.01, *** p < 0.001).

Figure 1 shows S_p_O_2_ during the different experimental conditions. PEEP-10 induced a significant increase in S_p_O_2_ compared to all conditions except PEEP-5. PEEP-5 also induced a significant increase in S_p_O_2_ compared to FB and PEEP-0. Compared to FB, the average increase in S_p_O_2_ was 5.6 ± 5.0% with PEEP-10 and 3.3 ± 4.6% with PEEP-5. Neither T10 nor PLB induced significant improvement in S_p_O_2_ compared to FB and PEEP-0. 

#### Quadriceps oxygenation

**Figure 2:**
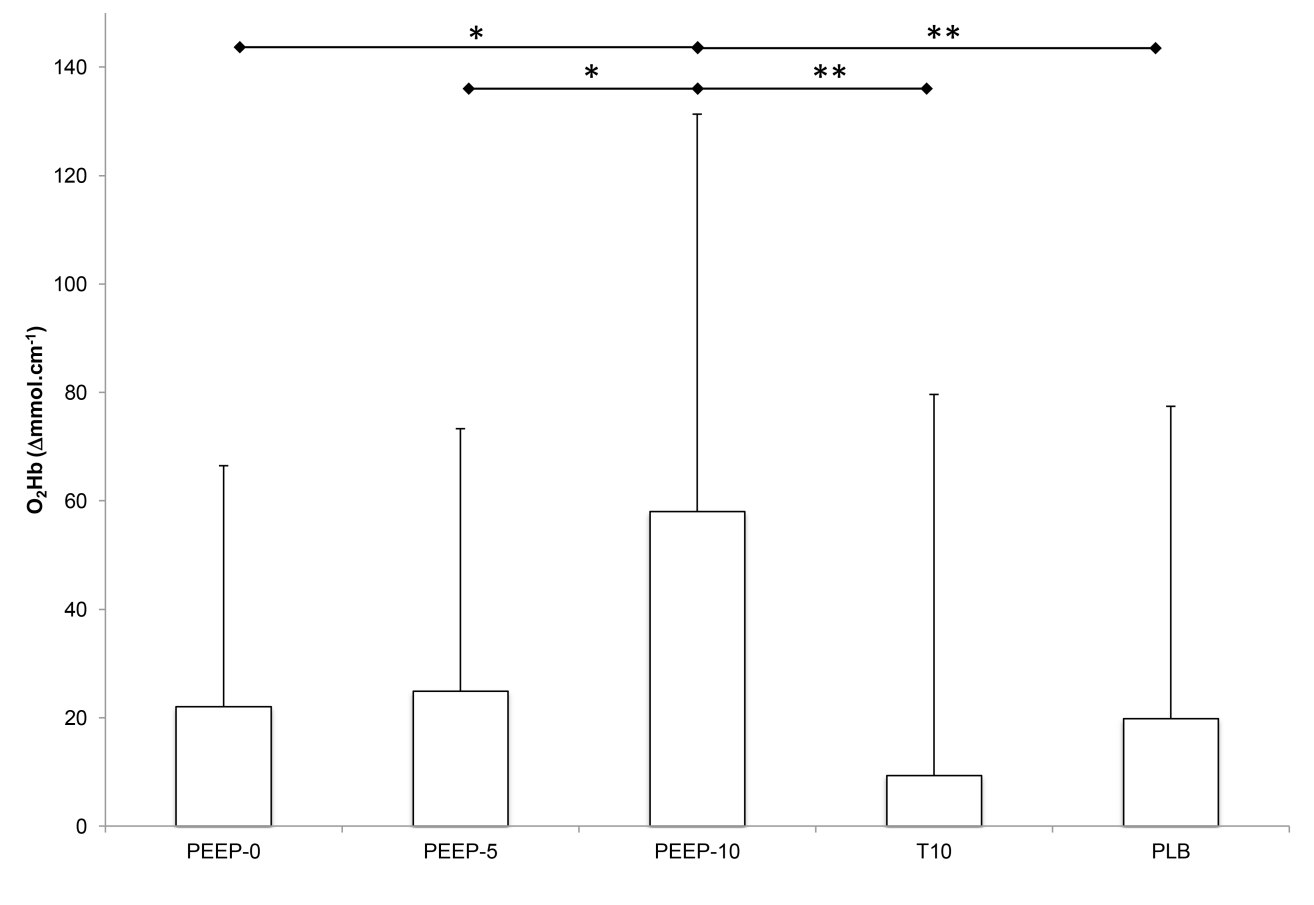
Changes in quadriceps oxyhemoglobin concentration (O_2_Hb, expressed as delta from the free breathing condition immediately before) in all experimental conditions. See Figure 1 for abbreviations. * significant difference between two conditions (*p < 0.05, **p < 0.01).

PEEP-10 only induced a significant increase in [O_2_Hb] compared to all the other conditions (Figure 2). No significant difference was observed in [HHb], [THb] and TOI between conditions (Table 1). 

**Table 1:** Changes in quadriceps oxygenation in all experimental conditions during the laboratory study.

	PEEP-0	PEEP-5	PEEP-10	T10	PLB
HHb (Δµmol·cm)	-13.6 (42.5)	-18.2 (51)	-43.3 (82.6)	-4.6 (68.7)	-14.6 (65.6)
THb (Δµmol·cm)	-3.3 (23.2)	-22.6 (12.6)	-42.6 (30.5)	1.9 (20.5)	-4.1 (39.3)
TOI (Δ%)	1.1 (3.3)	1.0 (2.5)	2.7 (4.2)	0.3 (3.8)	0.6 (3.5)
TOI (%)	67.4 (6.0)	68.1 (4.9)	69.1 (5.7)	67.9 (6.5)	66.8 (5.0)

Values are means (SD). All deltas values are relative to the free breathing condition immediately before. PEEP-0/5/10, Positive end expiratory pressure at 0, 5 and 10 cmH_2_O; T10, minute ventilation target corresponding to the spontaneous level measured in PEEP-10; PLB, pursed lips breathing; HHb, deoxyhemoglobin; THb, total hemoglobin; TOI, tissue oxygenation index (as changes from free breathing, i.e. Δ%, or in absolute value, i.e. %).

#### Ventilation

**Figure 3:**
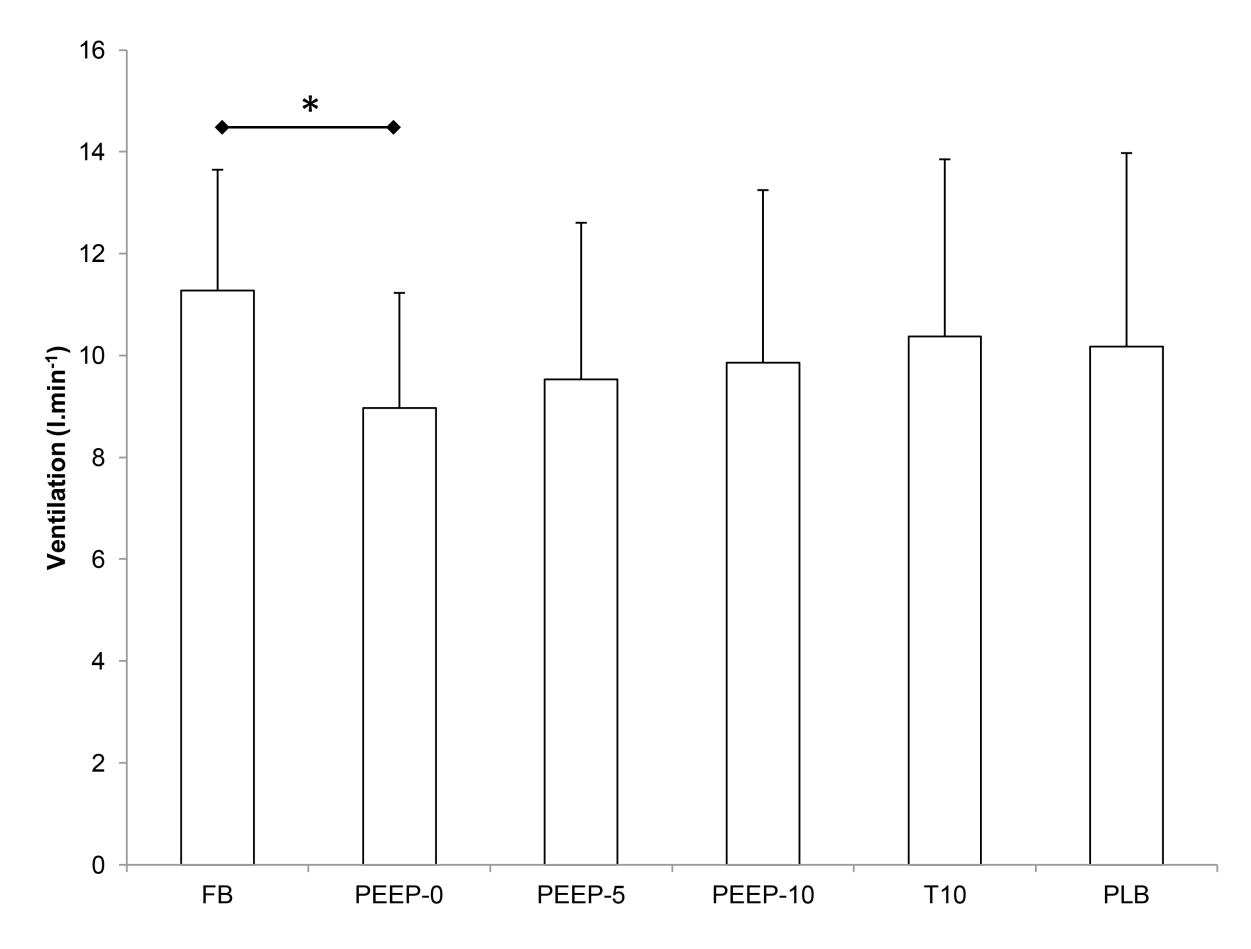
Minute ventilation in all experimental conditions. See Figure 1 for abbreviations. * significant difference between two conditions (p < 0.05).

Minute ventilation was larger in FB compared to PEEP-0 only (Figure 3). No other difference was observed between the experimental conditions. Table 2 shows end-tidal carbon dioxide partial pressure (P_ET_CO_2_) and breathing pattern in all experimental conditions. P_ET_CO_2_ was significantly larger in FB compared to all the other experimental conditions. Tidal volume was increased significantly with PEEP-10, T10 and PLB compared to FB while PLB only induced significantly larger tidal volume compared to PEEP-0. Breathing frequency was significantly larger in FB compared to all other experimental conditions while PLB only induced significantly lower values compared to PEEP-0. Expiratory time was smaller in FB compared to all other experimental conditions and it was also higher with PLB compared to all other conditions. Inspiratory time did not differ between conditions.

**Table 2:** End-tidal carbon dioxide partial pressure, breathing pattern, expiratory esophageal and gastric pressures and subjects’ subjective perception in all experimental conditions during the laboratory study.

	FB	PEEP-0	PEEP-5	PEEP-10	T10	PLB
P_ET_CO_2_ (mmHg)	37.1 (3.0)	35.7 (3.4)*	34.7 (3.9)*	34.6 (4.3)*	35.0 (4.5)*	36.4 (4.7)*
Vt (l)	0.83 (0.34)	0.93 (0.34)	0.94 (0.37)	1.07 (0.37)*	1.00 (0.51)*	1.26 (0.64)*^$^
fR (/min)	15.3 (4.9)	11.4 (3.8)*	11.5 (3.9)*	10.4 (4.0)*	11.7 (4.3)*	8.0 (4.8)*^$^
Ti (s)	2.1 (1.0)	2.5 (1.0)	2.2 (1.1)	2.4 (1.2)	2.8 (1.3)	2.5 (1.1)
Te (s)	2.5 (1.3)	3.5 (1.4)*	3.5 (1.6)*	4.0 (1.6)*	3.5 (1.5)*	8.1 (4.66)*^$^
P_es_ int expi (cmH_2_O·s·min^-1^)	5.7 (6.1)	10.4 (9.2)	14.9 (7.6)	22.8 (26.0)*^£^	3.8 (8.0)	45.1 (40.0)*^$£^
P_ga_ int expi (cmH_2_O·s·min^-1^)	24.1 (14.7)	33.9 (14.1)	44.7 (23.8)	60.5 (23.1)*	28.4 (12.6)^£^	110.4 (96.9)*^$£^
VAS ‘well being’	1.5 (1.9)	1.7 (2.0)	1.5 (1.5)	1.4 (1.6)	1.8 (1.8)	1.6 (2.0)
VAS ‘breathing effort’	0.8 (1.2)	1.2 (1.4)	2.0 (1.6)	2.3 (1.8)	1.3 (1.3)	1.4 (1.1)

Values are means (SD). P_ET_CO_2_, end-tidal carbon dioxide partial pressure; Vt, tidal volume; FB, breathing frequency; Ti, inspiratory duration; Te, expiratory duration; P_es_ int expi, integral of the esophageal pressure during expiration; P_ga_ int expi, integral of the gastric pressure during expiration; See Table 1 for other abbreviations. * significant difference compared to FB; **^*$*^** significant difference compared to PEEP-0; **^*£*^** significant difference compared to T10 (p < 0.05).

#### Esophageal and gastric pressures

**Figure 4:**
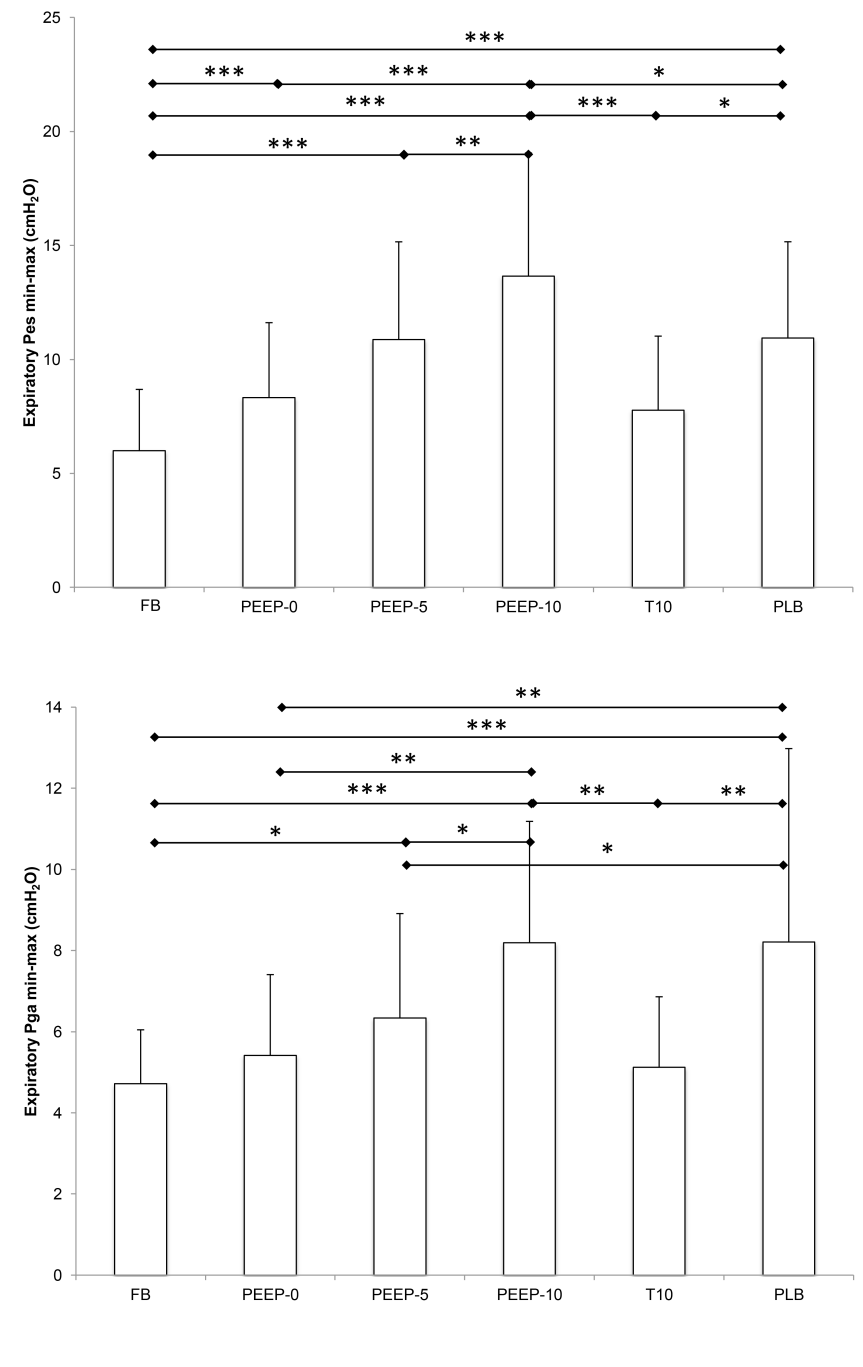
Expiratory esophageal and gastric pressure swing (P_es_ min-max and P_ga_ min-max) in all experimental conditions. See Figure 1 for abbreviations. * significant difference between two conditions (*p < 0.05, **p < 0.01, ***p < 0.001).

Integrated expiratory P_es_ and P_ga_ and amplitudes of expiratory P_es_ and P_ga_ swings during the experimental conditions are showed in Table 2 and Figure 4, respectively. The integrals of expiratory P_es_ and P_ga_ were significantly larger in PEEP-10 and PLB only compared to FB. PEEP-10 induced larger expiratory P_es_ swing compared to all the other conditions. PLB, PEEP-5 and PEEP-0 also induced larger expiratory P_es_ swing compared to FB. PEEP-10 and PLB induced larger expiratory P_ga_ swing compared to all the other conditions.

#### Cardiac function

Measurements of cardiac variables are shown in Table 3. All PEEP levels and T10 induced a significant decrease in HR compared to FB, without significant difference between PEEP, PLB and T10 conditions. SV was similar in all experimental conditions. Cardiac output was significantly larger in FB compared to all other conditions that did not differ between each other. 

**Table 3:** Cardiac function variables in all experimental conditions during the laboratory study.

	FB	PEEP-0	PEEP-5	PEEP-10	T10	PLB
HR (bpm)	68.9 (8.5)	65.9 (9.4)*	65.2 (10.0)*	64.8 (10.3)*	65.1 (9.9)*	66.9 (9.3)
Stroke volume (ml)	70.8 (20.0)	69.2 (17.5)	69.4 (16.0)	71.7 (14.8)	69.9 (15.9)	68.0 (16.2)
Q_C_ (l·min^-1^)	4.88 (1.48)	4.50 (1.06)*	4.44 (0.92)*	4.56 (0.79)*	4.49 (0.95)*	4.51 (1.08)*

Values are means (SD). HR, heart rate; Q_C_, cardiac output; See Table 1 for other abbreviations. * significant difference compared to FB (p < 0.05).

#### Subject’s perception

During the whole experimental session, no significant change in subjects’ perception of ‘breathing effort’ and ‘well-being’ was obtained (Table 2).

#### Correlations

The increases in O_2_Hb and S_p_O_2_ from FB to PEEP-10 were significantly correlated (r = 0.71, p = 0.02). No other significant correlation was found between individual changes in oxygenation (S_p_O_2_ or O_2_Hb) and changes in ventilation, P_es_, cardiac function and subjects’ perception for any condition (all r < 0.35 and p > 0.05).

### Field study

**Figure 5:**
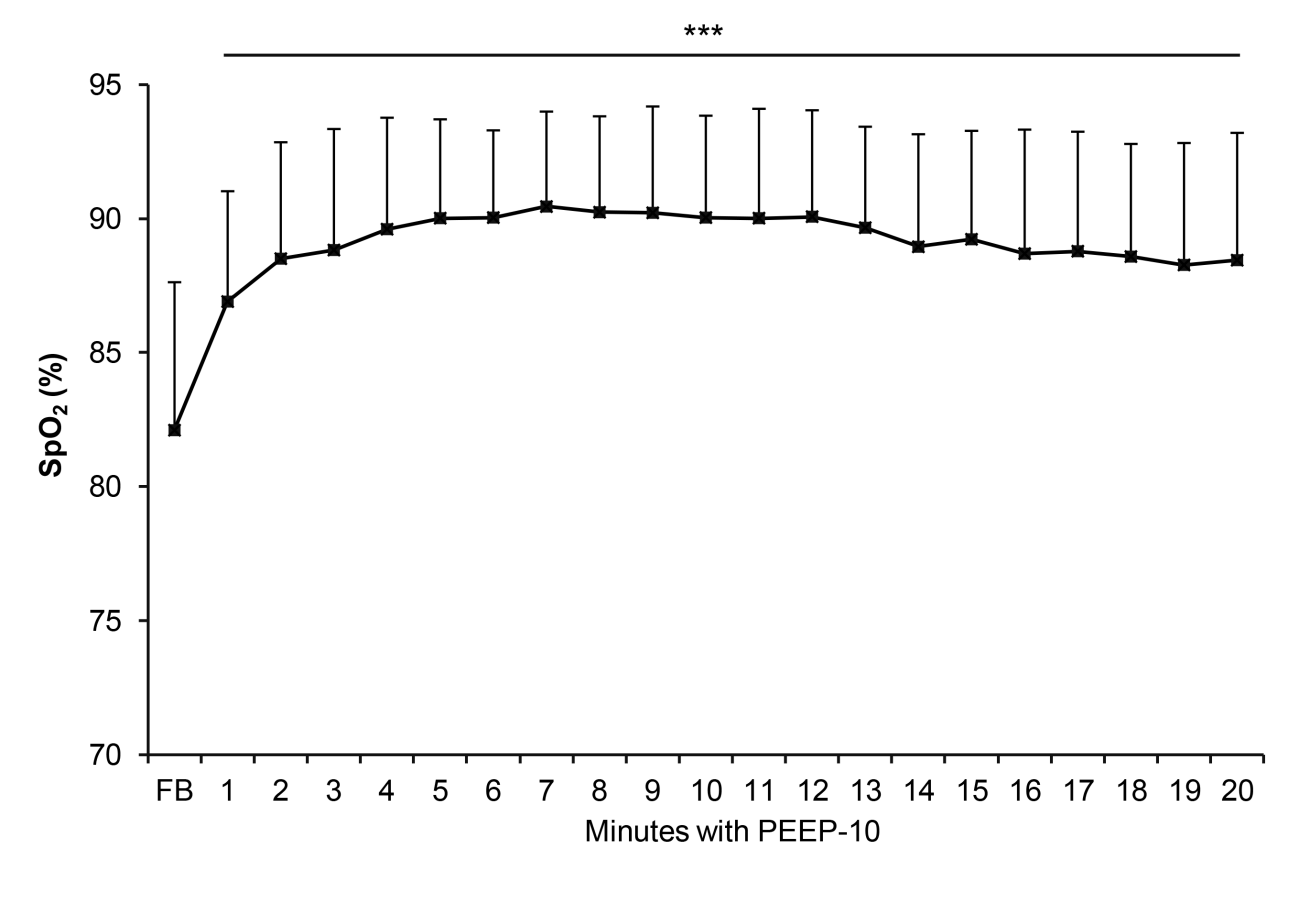
Arterial oxygen saturation (SpO_2_) during free breathing (FB) and during 20 min of PEEP-10 breathing at 4,350m of altitude. * significantly different compared to FB (*** p < 0.001).

At high altitude, PEEP-10 induced a significant increase in S_p_O_2_ compared to FB (Figure 5). S_p_O_2_ increased by 6.7 ± 6.0% from FB to the 3^rd^ minute with PEEP-10, with no further significant improvement until the 20^th^ minute with PEEP-10. 

## Discussion

The present findings demonstrate that using a 10-cmH_2_O PEEP in healthy subjects under normobaric (F_i_O_2_ = 0.12) and hypobaric (4,350 m) hypoxia induced a significant increase in S_p_O_2_ (+5.6% and +6.7% on average after 3 min PEEP-10 compared to FB, respectively). Measurements in the laboratory show that PEEP-10 also increased quadriceps oxygenation. This improved oxygenation was accompanied by a significant increase in expiratory P_es_ but with no changes in minute ventilation, breathing pattern or P_ET_CO_2_ compared to PEEP-0. No impairment in cardiac function was observed with increasing levels of PEEP and subjects did not report significant discomfort with positive expiratory pressure. These data suggest that breathing with a 10-cmH_2_O PEEP is an efficient non-pharmacological intervention to significantly improve arterial and muscle oxygenation under hypoxic conditions. This happens without substantial or deleterious changes in ventilation, cardiac function and subjects’ subjective perception of comfort. Conversely, PLB did not appear to provide similar positive effects on oxygenation.

Our results showed that PEEP-10 was the most efficient experimental condition to improve both S_p_O_2_ and muscle oxygenation (O_2_Hb) in healthy subjects exposed to hypoxia (Figures 1 and 2). Arterial and tissue oxygenation improvements were correlated. Even if quadriceps TOI was not significantly increased, a tendency for larger values with PEEP-10 was observed (p = 0.1) and further suggests an improvement in tissue oxygenation. While in the laboratory study the effects of PEEP breathing were measured for 3 min periods only, the field study showed that the SpO_2_ increase with PEEP-10 plateaued after 3 to 5 min (no further significant increase in SpO_2_ was observed after 3 min PEEP-10) and was maintained for up to 20 min (Figure 5). Importantly, the present results also show that PEEP-10 increases arterial oxygenation similarly during both normobaric and hypobaric hypoxic conditions despite their potential differences as recently emphasized [24]. 

The laboratory study showed that the oxygenation changes with PEEP-10 were observed without any increase in minute ventilation compared to PEEP-0, while PEEP conditions induced slightly lower minute ventilation compared to FB. Schoene et al. [15] have previously reported similar (in subjects with high-altitude pulmonary edema) or larger (in healthy subjects) minute ventilation with PEEP-10 compared to PEEP-0 at 4,400 m. Changes in minute ventilation with PEEP may depend on psychological factors (in the present study subjects were blinded for the PEEP level), subjects’ health status [15] or some PEEP device effects (as suggested by the significant difference between PEEP-0 and FB). Although no significant difference in breathing pattern was observed between PEEP conditions, PEEP-10 tended to induce deeper and slower breathing compared to PEEP-0. Such a breathing pattern may improve gas exchange and therefore potentially contribute to higher S_p_O_2_. However, the T10 condition that reproduced the same breathing pattern as PEEP-10 did not induce any improvement in blood or muscle oxygenation compared to PEEP-0 or FB, demonstrating that slight changes in breathing pattern associated with PEEP-10 are not the reasons for improved oxygenation. Hence, the improved oxygenation with PEEP-10 is not the consequence of changes in minute ventilation or breathing pattern as previously suggested [15].

P_es_, an index of pleural and intrathoracic pressures, was significantly higher with PEEP-10 compared to other PEEP conditions or FB. This P_es_ increase suggests that alveolar pressure was enhanced with PEEP-10 and this could have played a major role regarding the increased S_p_O_2_. In case of an early pulmonary sub-edema, as suggested by Agostoni et al. [11], PEEP-10 may resorb some of the extra-vascular fluid accumulation and as a consequence improve oxygen diffusion. We recently reported significant extravascular fluid accumulation in healthy subjects during the first days of high altitude exposure similar to the present field study [25]. While some reduction in extravascular fluid accumulation due to PEEP-10 may explain the improvement in arterial oxygenation in our field study, the presence of extravascular fluid accumulation during a short duration hypoxic exposure as in the laboratory study (< 2 h) remains however hypothetical. Other mechanisms such as increased operational lung volume and improved ventilation/perfusion matching (especially by increasing ventilation in lung regions with low ventilation/perfusion ratio) may underlie the improved gas exchange and arterial oxygenation observed in the present study.

An increase in intrathoracic and abdominal (as shown by P_ga_ measurement in the present study) pressure is also known to decrease the venous return and therefore to have potential deleterious effects on cardiac function [17]. However, in the present study, the absence of change in SV estimated by impedance cardiography suggests that applying a 10-cmH_2_O PEEP under resting conditions had no major consequences on venous return and left cardiac function. HR and consequently Qc were slightly but significantly reduced during all PEEP conditions compared to FB. The underlying mechanisms of this device-effect remain to clarify but may be, at least in part, related to the slight differences in ventilation observed between PEEP conditions and FB. Expiratory phases were longer in PEEP conditions compared to FB and it is known that respiratory-circulatory interactions such as respiratory sinus arrhythmia by which the ECG R-R interval is shortened during inspiration and prolonged during expiration (i.e., reduced HR), are part of the efficiency of pulmonary gas exchanges [26]

No significant discomfort was reported by the subjects when using PEEP as indicated by the levels of ‘breathing effort’ and ‘well-being’ scored all over the protocol. These results indicate that using PEEP even at 10 cmH_2_O in healthy subjects does not induce any deleterious perceptions and is well tolerated both under simulated or real altitude conditions. 

PEEP-5 also induced a significant increase in S_p_O_2_ compared to both FB and PEEP-0 conditions, but no change in quadriceps oxygenation was induced. The relevance of this improvement in arterial oxygenation is questionable since the average increase in S_p_O_2_ induced by PEEP-5 (3.3%) was clearly below the 4.9% S_p_O_2_ difference previously reported to distinguish subjects with or without symptoms at altitude [8]. P_es_ measurements indicate that PEEP-5 induced significantly lower increase in intrathoracic pressure and possibly in alveolar pressure compared to PEEP-10. Conversely, PEEP-5 had similar effects than PEEP-10 on ventilation and cardiac variables. Hence, we suggest that PEEP-5 does not sufficiently increase intrathoracic pressure to promote relevant improvement in both S_p_O_2_ and O_2_Hb as observed with PEEP-10. 

In our laboratory study assessing for the first time the effect of PLB in a group of healthy subjects under normobaric hypoxic condition, PLB did not induce any significant change in S_p_O_2_ and quadriceps oxygenation. These results are in contrast to the case study by Tannheimer et al. [20] reporting a large S_p_O_2_ increase in a subject with high altitude pulmonary edema at 4,330 m after 30 min of PLB. An important difference between Tannheimer’s study and ours is that we investigated healthy subjects during a short-duration hypoxic exposure with 3-min phases whereas PLB was investigated after a 4-day rapid ascent and during 30 min in Tannheimer’s study. Also, S_p_O_2_ during FB was higher in our subjects (78 ± 6% with FiO_2_ = 0.12) compared to Tannheimer’s case report (62%) where the potential for an improvement in S_p_O_2_ was larger. Moreover, expiratory pressure and minute ventilation were not measured in this previous study, making comparisons with the present work difficult. The specific changes induced by PEEP-10 compared to PLB regarding breathing pattern (expiratory duration twice longer with PLB) and P_es_ (greater integrated expiratory P_es_ but lower expiratory P_es_ swing with PLB) may explain the absence of oxygenation improvement with PLB.

Several limitations of the present study should be underlined. First, in the laboratory study each experimental condition was tested for 3-min periods only. The field study showed however that most of the SpO_2_ change with PEEP-10 observed over 20 min occurs within this time frame. In addition, physiological variables measured during the laboratory study reached steady states during all 3-min phases and repeating all conditions in a randomized order two times allowed us to verify the absence of any incidental effect on the main outcomes. The laboratory study was performed during a short hypoxic exposure duration (i.e. <2 h) while the field study was performed at least 2 days after arrival at 4,350 m of altitude. This has two important consequences. First, laboratory and field hypoxic conditions were associated with different states of physiological adaptation to hypoxia. The present results suggest however that PEEP has similar effect on arterial oxygenation despite differences in prior hypoxic exposure duration in the laboratory and field studies, which is in contrast with previous results suggesting that the effect of continuous positive airway pressure on arterial oxygen saturation may depend on the time spent at high altitude [11]. Second, it is well known that AMS normally appears within 8 to 48 h after arrival at high altitude. Therefore, since the subjects in the laboratory had no AMS symptoms while those at altitude had no more significant AMS symptoms after the initial 2 days of acclimatization (Lake Louise score < 3 [27]), the present study cannot evaluate whether PEEP may improve AMS symptoms and acclimatization to high altitude. Further studies are needed to clarify the influence of previous hypoxic exposure duration on the effect of PEEP and to evaluate its ability to reduce AMS symptoms. At last, it should be acknowledged that impedance cardiography and NIRS are indirect measurements of cardiac output and tissue oxygenation, respectively. The measurement of cardiac output with the Physioflow® system has been validated against the direct Fick method [22]. Although NIRS measurement displays important limitations such as the relatively superficial portion of the tissue assessed and the potential influence of superficial layers (skin, subcutaneous fat, etc) [28], it provides in the present study a reliable estimate of relative changes in muscle hemoglobin oxygenation and oxygenation index between free breathing and PEEP breathing conditions.

To conclude, our study shows for the first time that PEEP-10 significantly improves both S_p_O_2_ and O_2_Hb in healthy subjects during hypoxic exposure, without significant changes in ventilation, impairments of cardiac function and subjects’ subjective perception of discomfort. The main physiological mechanism promoting improved oxygenation may be the increased intrathoracic and consequently alveolar pressures induced by PEEP-10, enhancing alveolo-capillary oxygen diffusion. The rapid ~6% average increase in S_p_O_2_ induced by PEEP-10 may be relevant to improve AMS symptoms [8] and could be an attractive non-pharmacological tool to prevent or treat high-altitude illnesses in combination with other interventions. 
